# Efficacy and safety of obinutuzumab in active lupus nephritis

**DOI:** 10.1093/ckj/sfaf161

**Published:** 2025-06-26

**Authors:** Corina-Daniela Ene, Annette Bruchfeld

**Affiliations:** Department of Nephrology and Internal Medicine, Faculty of General Medicine, Carol Davila University of Medicine and Pharmacy, Bucharest, Romania; Dr Carol Davila Clinical Hospital of Nephrology, Bucharest, Romania; Department of Health, Medicine and Caring Sciences, Linköping University, Linköping, Stockholm, Sweden; Department of Renal Medicine, Karolinska University Hospital and CLINTEC Karolinska Institutet, Stockholm, Sweden

Lupus nephritis (LN) is one of the most frequent manifestations of systemic lupus erythematosus (SLE), and has an impact on long-term kidney function and patient prognosis. SLE and LN pathogenesis is marked by dysregulation of B- and T-cell responses, autoantibodies overproduction against self-antigens and complement activation, leading to immune complex deposition in the kidneys, glomerular damage and renal fibrosis. Although the therapeutic armamentarium has evolved considerably over recent years, including the regulatory approvals of belimumab in 2020 and voclosporin in 2021, short- and long-term renal outcomes remain not satisfactory [1]. B-cell depletion has been proposed as a potential therapeutic target in LN. Anti-CD20 monoclonal antibodies (mAbs), are approved for the treatment of B-cell proliferative disorders, including non-Hodgkin's lymphoma, and several auto-immune disorders such as rheumatoid arthritis and anti-neutrophil cytoplasmic antibody (ANCA) vasculitis.

Rituximab, a type I chimeric anti-CD20 mAb, which depletes B cells via complement-dependent cytotoxicity and antibody-dependent cellular cytotoxicity/phagocytosis (ADCC/ADCP), was evaluated as an add-on to standard of care in the LUNAR (Lupus Nephritis Assessment with Rituximab) trial but showed no significant improvement in renal response compared with placebo [2]. Obinutuzumab, a humanized glycoengineered type II anti-CD20 mAb approved for the treatment of chronic lymphocytic leukemia and follicular lymphoma, was developed to enhance ADCC and ADCP relative to rituximab leading to more profound and sustained B-cell depletion. Nobility, a phase 2, randomized, placebo-controlled trial demonstrated improvements in complete renal response (CRR) [defined as composite measure requiring urinary protein-to-creatinine ratio (UPCR) <0.5, normal renal function without worsening of baseline serum creatinine by >15% and inactive urinary sediment], and overall renal responses at Weeks 52, 76 and 104 compared with those receiving placebo and standard of care (SOC) [3]. A post-hoc analysis furthermore demonstrated a favourable impact of obinutuzumab on reductions in estimated glomerular filtration rate (eGFR) decline and LN flares, and more patients who achieved CRR at Week 76 with obinutuzumab were able to maintain a daily prednisone dose of 7.5 mg suggesting a glucocorticoid-sparing effect [4].

The Regency trial, a phase 3, randomized, double-blind, placebo-controlled trial, evaluated the efficacy and safety of obinutuzumab in patients with active biopsy-proven LN in 15 countries [5]. The inclusion and exclusion criteria for the trial are presented in Table 1. Patients were randomized in a 1:1 ratio and assigned to the intervention arm receiving obinutuzumab in one of the two dose schedules (Table 1) or in the control arm. All subjects received SOC with mycophenolate mofetil and prednisone before or at randomization (Table 1). The primary endpoint was CRR at Week 76 (definition Table 1), while the secondary endpoints included a CRR at Week 76 with 7.5 mg prednisone per day, partial renal response, changes in eGFR and patient-reported fatigue (Table 1) in a 1:1 ratio [5].

**Table 1:** Methods of REGENCY trial.

Inclusion criteria	Age 18–75 years
	Class III/IV ± concomitant class V on renal biopsy during or within 6 months before screening
	A UPCR ≥1 g (from 24 h proteinuria)
	ANA titre of ≥1:80 on HEp-2 cells or ≥1 equivalent positive ANA test
Exclusion criteria	eGFR <30 mL/min/1.73 m^2^
	End-stage renal disease (dialysis/transplantation)
	Active infection
	anti-CD20 therapy in the last 9 months
	Other immunosuppressive therapies in the last 2 months (cyclophosphamide, tacrolimus, cyclosporine, voclosporin)
Randomization	1:1 ratio to receive obinutuzumab or placebo, stratified by geographic region and race
Intervention	Obinutuzumab—1000 mg on Day 1, at Weeks 2, 24, 26 and 52
	With/without additional dose at Week 50
	SOC—mycophenolate mofetil
	Prednisone—7.5 mg per day by Week 12 and 5 mg per day by Week 24
Primary endpoint	CRR at Week 76: proteinuria <0.5 mg/mg, eGFR ≥85% of baseline (calculated by 2009 CKD-EPI), no intercurrent events (rescue therapy, treatment failure, death or early trial withdrawal)
Secondary endpoints	Complete proteinuric response at week, defined as UPCR lower than 0.8 at Week 76 with no intercurrent event
	Complete renal response with successful prednisone tapper at Week 76–prednisone dose >7.5 mg per day or lower between Weeks 64 and 76
	Renal-related events (death, treatment failure, worsening proteinuria, worsening eGFR) or death
	Change in the eGFR from baseline to Week 76
	Overall renal response at Week 50 defined as a complete renal response or a partial renal response^a^—change in anti-dsDNA, C3, C4 from baseline to Week 50
	Change in score on the Functional Assessment of Chronic Illness Therapy–Fatigue

^a^A partial renal response was defined as following: at least a 50% reduction in the UPCR from baseline, a UPCR <1, an eGFR of at least 85% of the baseline value and no occurrence of an intercurrent event.

CKD-EPI, Chronic Kidney Disease Epidemiology Collaboration.

**Figure 1:**
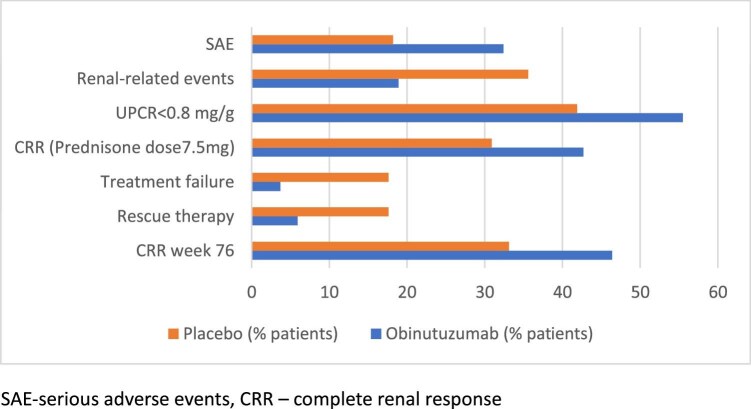
Primary and key secondary endpoint results in REGENCY trial. SAE, serious adverse events.

A total of 513 patients were screened—271 were randomized to obinutuzumab (*n* = 135) or placebo (*n* = 136) groups, both groups with similar characteristics. The primary endpoint was achieved in 46.4% of the obinutuzumab group compared with 33.1% of the placebo group [5]. Regarding the secondary endpoints, the CRR in subjects receiving 7.5 mg/day, and the reduction of UPCR <0.8 without an intercurrent event were significantly higher in the obinutuzumab arm compared with placebo, while the eGFR endpoint did not reach statistical significance (Fig. 1). The serological and pharmacodynamic analyses from baseline to Week 76 indicated greater adjusted mean changes in levels of C3 complement, C4 complement and antibodies against dsDNA in the obinutuzumab group compared with placebo group. The percentage of patients with peripheral B-cell depletion over time, defined by an absolute CD19-positive B-cell count, was higher in obinutuzumab group compared with placebo. No unexpected safety signals were identified. The most frequent adverse events were infections, drug induced neutropenia and events related to severe acute respiratory syndrome coronavirus 2 (SARS-CoV-2) infection were more prevalent in the obinutuzumab arm (Fig. 1) [5].

This trial confirms the efficacy of obinutuzumab on top of standard therapy in achieving complete renal response, placing type 2 anti-CD20 antibodies as potential future therapeutic agent for LN. However, questions remain regarding the infection risk, long-term safety and renal outcome of obinutuzumab, which merits further study.
